# Genome-Wide Reverse Genetics Framework to Identify Novel Functions of the Vertebrate Secretome

**DOI:** 10.1371/journal.pone.0000104

**Published:** 2006-12-20

**Authors:** Michael A. Pickart, Eric W. Klee, Aubrey L. Nielsen, Sridhar Sivasubbu, Eric M. Mendenhall, Brent R. Bill, Eleanor Chen, Craig E. Eckfeldt, Michelle Knowlton, Mara E. Robu, Jon D. Larson, Yun Deng, Lisa A. Schimmenti, Lynda B.M. Ellis, Catherine M. Verfaillie, Matthias Hammerschmidt, Steven A. Farber, Stephen C. Ekker

**Affiliations:** 1 Department of Genetics, Cell Biology and Development, University of Minnesota, Minneapolis, Minnesota, United States of America; 2 Arnold and Mabel Beckman Center for Transposon Research, University of Minnesota, Minneapolis, Minnesota, United States of America; 3 Department of Oral Sciences and Minnesota Craniofacial Research Training Program MinnCResT, University of Minnesota, Minneapolis, Minnesota, United States of America; 4 Department of Pediatrics, Genetics and Metabolism and Department of Ophthalmology, University of Minnesota, Minneapolis, Minnesota, United States of America; 5 Laboratory Medicine and Pathology and Computer Science and Engineering, University of Minnesota, Minneapolis, Minnesota, United States of America; 6 Department of Medicine, Division of Hematology, Oncology, and Transplantation, and Stem Cell Institute, University of Minnesota, Minneapolis, Minnesota, United States of America; 7 Carnegie Institute of Washington, Baltimore, Maryland, United States of America; 8 Max Planck Institute Immunbiologie, Freiburg, Germany; University of Maryland, United States of America

## Abstract

**Background:**

Understanding the functional role(s) of the more than 20,000 proteins of the vertebrate genome is a major next step in the post-genome era. The approximately 4,000 co-translationally translocated (CTT) proteins – representing the vertebrate secretome – are important for such vertebrate-critical processes as organogenesis. However, the role(s) for most of these genes is currently unknown.

**Results:**

We identified 585 putative full-length zebrafish CTT proteins using cross-species genomic and EST-based comparative sequence analyses. We further investigated 150 of these genes (Figure 1) for unique function using morpholino-based analysis in zebrafish embryos. 12% of the CTT protein-deficient embryos resulted in specific developmental defects, a notably higher rate of gene function annotation than the 2%–3% estimate from random gene mutagenesis studies.

**Conclusion(s):**

This initial collection includes novel genes required for the development of vascular, hematopoietic, pigmentation, and craniofacial tissues, as well as lipid metabolism, and organogenesis. This study provides a framework utilizing zebrafish for the systematic assignment of biological function in a vertebrate genome.

## Introduction

**Figure 1:**
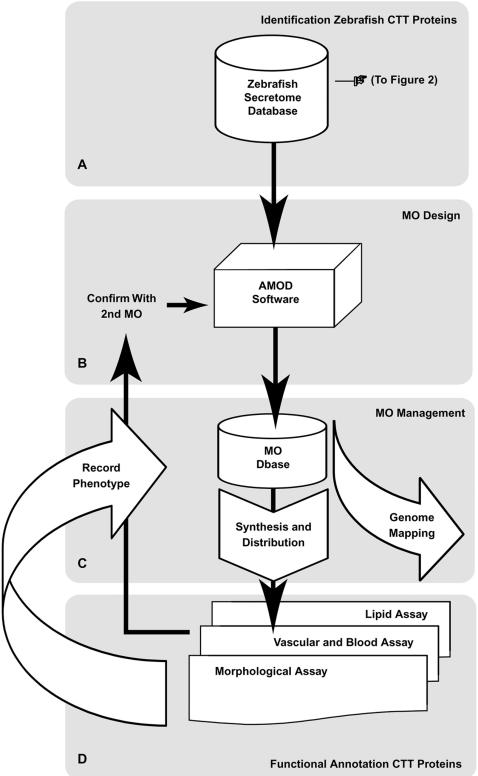
Schematic of overall MO screen. (A) A bioinformatics pipeline was developed to determine the subset of N-terminally complete CTT homologs representing the testable putative proteins of the zebrafish secretome. (B) AMOD software was developed to standardize and increase the efficiency of the MO design process to allow more rapid screening. (C) A MO database, MODB, was developed to manage, share, and data mine all MO design and outcome information. (D) Following MO synthesis and distribution to the participating labs, MOs were investigated using a variety of assays in zebrafish embryos that allowed functional annotation of 18/150 of the putative CTT proteins investigated. Results of investigations were recorded in MODB for data mining. AMOD software was used to design a second sequence-independent MO to assess specificity of the initial MO tested.

**Figure 2:**
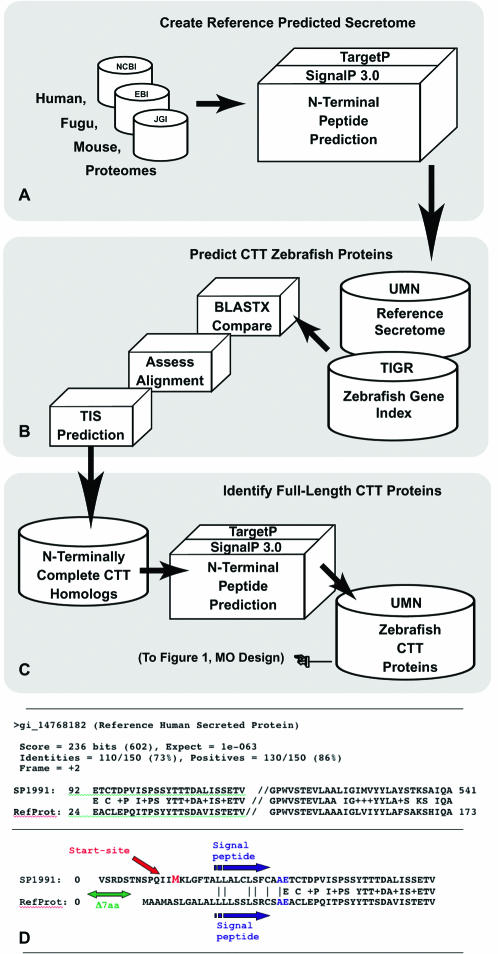
Selection of candidate genes for MO targeting. (A) Co-translationally translocated vertebrate protein sequences (CTT Proteins) were identified using an *in silico* prediction pipeline to create the reference CTT protein sequence sets. (B) Zebrafish Tentative Consequence (TC) sequences were compared to the reference protein sequence sets using BLASTX. Zebrafish TC sequences possessing highly homologous regions located near the reference protein sequence N-termini and possessing clear translational initiation sites were selected for further analysis. (C) The corresponding zebrafish TC peptides were then analyzed by the *in silico* prediction pipeline and sequences possessing a signal peptide selected for morpholino design. (D) E.g. zebrafish sequence SP1991 was selected on the basis of its strong homology to the N-terminus of reference protein gi_14768182. Analysis identified a strong translation initiation site near the 5′ end of the SP1991 nucleotide sequence and *in silico* predictions identified a clear signal peptide near the N-terminus of the translated peptide.

The increasing availability of genomic and EST sequence data for model genetic organisms has greatly facilitated genome-wide approaches for gene discovery and analysis. We used a morpholino-based gene ‘knockdown’ strategy (Figure 1) to assess the role of members of the secretome in vertebrate development and function[1]. A software pipeline (Figure 2) for comparative genomic data mining was developed to identify CTT proteins en route to the endoplasmic reticulum, cell membranes, or external regulatory sites[2], [3]. Utilizing the TargetP and SignalP algorithms for signal peptide and cleavage site prediction, reference CTT protein sets were created from completed genome projects (*H. sapiens*, *F. rubripes*, and *M. musculis*) and compared to sequence data from the TIGR Zebrafish Gene Indices (versions 6.1, 8, 12, and 16) and the Zebrafish Genome Project (Sanger, build Zv2 – Zv6) to identify putative CTT proteins for reverse genetic analysis. To overcome the 3′ bias of most EST sequence information, the combined comparative analysis and secreted protein predictive software ensured that target proteins selected possessed N-terminally complete sequence information.

Morpholino phosphorodiamidate oligonucleotides (MOs), neutrally charged nucleic acid analogs created by replacing the ribose sugar with a morpholine moiety and the phosophodiester backbone with a phosphorodiamidate linkage[4], were used to target the putative 585 CTT proteins identified for loss-of-function studies in zebrafish embryos[1]. The translational initiation site (TIS) of the respective CTT-coding sequence was identified with the assistance of AMOD, MO design software created for these studies[3]. The use of MO-based reversed genetics necessitates TIS identification as MOs are most effective through Watson-Crick base pairing of RNA target sequences at or upstream (5′) of the TIS. AMOD-assisted design helped to ensure target sequences were chosen with appropriate properties of efficacy and uniqueness of target sequence.

## Results

Following injection of MOs against CTT proteins by the joint effort of multiple laboratories, we observed distinct developmental phenotypes in 18 of the first 150 genes investigated using this approach. Each of the research partners have contributed different zebrafish screening approaches resulting in the novel observations summarized here. The complete results of the ongoing screen have been stored in the Arnold and Mabel Beckman Center for Transposon Research Morpholino Database (MODB, http://www.secretomes.umn.edu/MODB/) for access to MO-specific information and phenotype data mining with the goal of accelerating the assignment of gene function to sequence. Criteria for inclusion in Table 1 include: 1) MO-induced effects were not accompanied by non-specific effects sometimes observed with MO treatment (unpublished observations), 2) phenotypes were observed in >50% of embryos injected at doses less than or equal to 5 ng, and 3) phenotypes were dependent on MO dose. In addition, 15/16 of the phenotypes in Table 1 were reproducible when two sequence-independent MOs were tested and, furthermore, these demonstrated synergistic effects when both MOs against the same target gene were injected together (criteria for specificity as in[5], [6]). Thus, the first three criteria above provide a high specificity threshold, i.e. predict that additional target sites reliably reproduce the initial phenotype. The current annotation based on multiple-species sequence homology for each of these genes (TIGR, Esemble Zv6) proscribes these genes to a variety of distinct proteins that are not from the same family or other similarity in functional or structural classification.

**Table 1:**
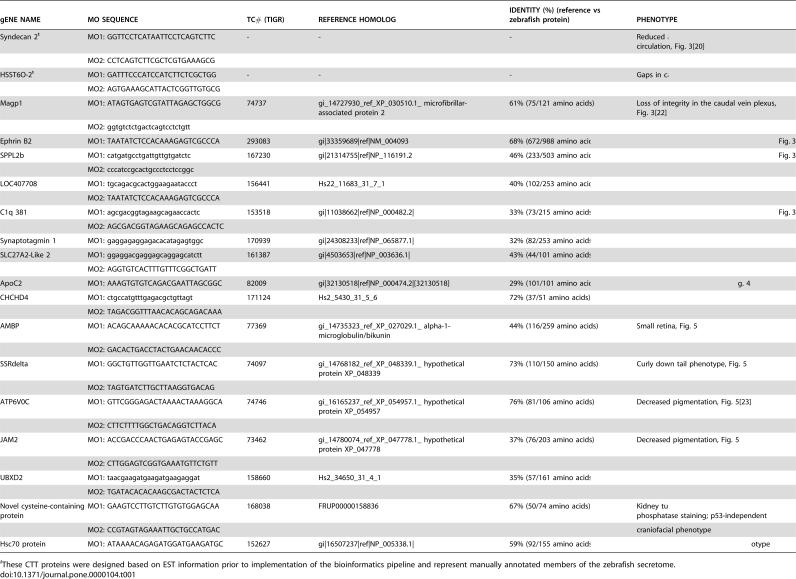


gENE NAME	MO SEQUENCE	TC# (TIGR)	REFERENCE HOMOLOG	IDENTITY (%) (reference vs zebrafish protein)	PHENOTYPE
Syndecan 2ŧ	MO1: GGTTCCTCATAATTCCTCAGTCTTC	-	-	-	Reduced axial or lack of intersegmental circulation, Fig. 3 [20]
	MO2: CCTCAGTCTTCGCTCGTGAAAGCG				
HSST6O-2ŧ	MO1: GATTTCCCATCCATCTTCTCGCTGG	-	-	-	Gaps in caudal vein, Fig. 3 [21]
	MO2: AGTGAAAGCATTACTCGGTTGTGCG				
Magp1	MO1: ATAGTGAGTCGTATTAGAGCTGGCG	74737	gi_14727930_ref_XP_030510.1_ microfibrillar-associated protein 2	61% (75/121 amino acids)	Loss of integrity in the caudal vein plexus, Fig. 3 [22]
	MO2: ggtgtctctgactcagtcctctgtt				
Ephrin B2	MO1: TAATATCTCCACAAAGAGTCGCCCA	293083	gi|33359689|ref|NM_004093	68% (672/988 amino acids)	Premature return of caudal vein blood flow, Fig. 3
SPPL2b	MO1: catgatgcctgattgttgtgatctc	167230	gi|21314755|ref|NP_116191.2	46% (233/503 amino acids)	Premature return of caudal vein blood flow, Fig. 3
	MO2: cccatccgcactgccctcctccggc				
LOC407708	MO1: tgcagacgcactggaagaataccct	156441	Hs22_11683_31_7_1	40% (102/253 amino acids)	Premature return of caudal vein blood flow, Fig. 3
	MO2: TAATATCTCCACAAAGAGTCGCCCA				
C1q 381	MO1: agcgacggtagaagcagaaccactc	153518	gi|11038662|ref|NP_000482.2|	33% (73/215 amino acids)	Premature return of caudal vein blood flow, Fig. 3
	MO2: AGCGACGGTAGAAGCAGAGCCACTC				
Synaptotagmin 1	MO1: gaggagaggagacacatagagtggc	170939	gi|24308233|ref|NP_065877.1|	32% (82/253 amino acids)	Decrease blood and pooling, Fig. 3
SLC27A2-Like 2	MO1: ggaggacgaggagcaggagcatctt	161387	gi|4503653|ref|NP_003636.1|	43% (44/101 amino acids)	Blood absent, Fig. 3
	MO2: AGGTGTCACTTTGTTTCGGCTGATT				
ApoC2	MO1: AAAGTGTGTCAGACGAATTAGCGGC	82009	gi|32130518|ref|NP_000474.2|[32130518]	29% (101/101 amino acids)	Poor BODIPY-C12 utilization, Fig. 4
CHCHD4	MO1: ctgccatgtttgagacgctgttagt	171124	Hs2_5430_31_5_6	72% (37/51 amino acids)	Otoliths absent, Fig. 5
	MO2: TAGACGGTTTAACACAGCAGACAAA				
AMBP	MO1: ACAGCAAAAACACACGCATCCTTCT	77369	gi_14735323_ref_XP_027029.1_ alpha-1-microglobulin/bikunin	44% (116/259 amino acids)	Small retina, Fig. 5
	MO2: GACACTGACCTACTGAACAACACCC				
SSRdelta	MO1: GGCTGTTGGTTGAATCTCTACTCAC	74097	gi_14768182_ref_XP_048339.1_ hypothetical protein XP_048339	73% (110/150 amino acids)	Curly down tail phenotype, Fig. 5
	MO2: TAGTGATCTTGCTTAAGGTGACAG				
ATP6V0C	MO1: GTTCGGGAGACTAAAACTAAAGGCA	74746	gi_16165237_ref_XP_054957.1_ hypothetical protein XP_054957	76% (81/106 amino acids)	Decreased pigmentation, Fig. 5 [23]
	MO2: CTTCTTTTGGCTGACAGGTCTTACA				
JAM2	MO1: ACCGACCCAACTGAGAGTACCGAGC	73462	gi_14780074_ref_XP_047778.1_ hypothetical protein XP_047778	37% (76/203 amino acids)	Decreased pigmentation, Fig. 5
	MO2: CTTGGAGTCGGTGAAATGTTCTGTT				
UBXD2	MO1: taacgaagatgaagatgaagaggat	158660	Hs2_34650_31_4_1	35% (57/161 amino acids)	Decreased pigmentation, Fig. 5
	MO2: TGATACACACAAGCGACTACTCTCA				
Novel cysteine-containing protein	MO1: GAAGTCCTTGTCTTGTGTGGAGCAA	168038	FRUP00000158836	67% (50/74 amino acids)	Kidney tubules defect as detected by alkaline phosphatase staining; p53-independent
	MO2: CCGTAGTAGAAATTGCTGCCATGAC				craniofacial phenotype
Hsc70 protein	MO1: ATAAAACAGAGATGGATGAAGATGC	152627	gi|16507237|ref|NP_005338.1|	59% (92/155 amino acids)	p53-independent craniofacial phenotype

ŧ These CTT proteins were designed based on EST information prior to implementation of the bioinformatics pipeline and represent manually annotated members of the zebrafish secretome.

**Figure 3:**
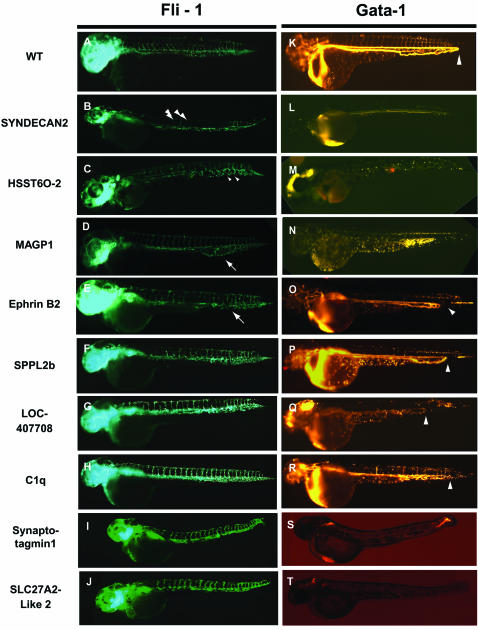
Defects in vasculogenesis and hematopoeisis observed in Tg (*fli-1:eGFP* (green)) or Tg (*gata-1:DsRed* (red)) embryos following MO inactivation of select CTT genes. (A) Normal vascular development observed in untreated Tg (fli-1:eGFP) embryos. (B, L) Decreases in the number of vascular sprouts (arrow heads) observed following injection of MO targeting Syndecan-2[20]. (C, M) Gaps within the caudal vein plexus (small arrow heads) observed following injection of MO targeting heparin sulfatetransferase-6-O 2-sulfotransferase (HSST6O-2)[21]. (D, N) Loss of integrity in the caudal vein plexus (arrow) observed following injection of MO targeting MAGP1[22]. Premature return in caudal vein flow shown by gata-1:dsRed expression to varying severities (arrowheads) following injection of MOs targeting Ephrin B2 (E,O), SPPL2b (F,P), predicted protein LOC407708 (G, Q), and C1q (H, R). Note: the premature return defects were not shown by fli-1:eGFP expression (E, F, G, H), however, were confirmed by other vascular markers (data not shown). (K) Normal blood development observed in untreated Tg (*gata-1:DsRed*) embryos. Decreased number of blood cells observed in 2 dpf embryos following injection with MO against Synaptotagmin13 (S) or Novel Protein similar to SLC27A2 (T). Accompanying panels (I) and (J) display no major vasculature defects for each of these genes respectively.

MO injection into vascular *fli-1*:*eGFP* and hematopoietic *gata-1:DsRed* double reporter transgenic (Tg) embryos (Figure 3A and 3K) resulted in a variety of specific developmental phenotypes without any accompanying gross morphologic effects (Figure 3). Reduced axial or a lack of intersegmental circulation was observed following injection of *syndecan-2* MO (Figure 3B). Observations of blood flow in the Tg (*gata-1*: *DsRed*) injected embryos confirmed these results as evidenced by an incomplete lack of circulation (Figure 3L). Injection of *heparin sulfatetransferase-6-O 2-sulfotransferase(HSST6O-2)*-MO produced gaps within the caudal vein plexus (Figure 3C, small arrow heads) that appeared to disrupt blood flow in Tg (*gata-1*: *DsRed*) injected embryos (Figure 3M). *MAGP1*-MO injected embryos resulted in a loss of integrity in the caudal vein plexus (Figure 3D, arrow) with disrupted blood flow (Figure 3N).

A group of related phenotypes characterized by a premature return of caudal vein blood flow was observed as a direct result of screening in Tg *(fli-1*:*eGFP*) and Tg (*gata-1:DsRed*) double transgenic embryos. MOs targeting *Ephrin B2* (Figure 3O, arrow), *SPPL2b* (Figure 3P, arrow), predicted protein *LOC407708* (Figure 3Q), and *C1q* (Figure 3R) resulted in embryos that failed to develop the more extended pattern of flow in the tail as compared to wild-type embryos (Figure 3K). Interestingly, the lack of complete flow was not a result of defects in gross vascular development as MO-injected Tg (*fli-1*:*eGFP*) embryos appeared normal (Figure 3E, 3F, 3G, and 3H, respectively) and lumenization was normal as determined by microangiography (data not shown).

The pattern of blood flow in Tg (*gata-1:DsRed*) embryos was also particularly useful in the identification of two new hematopoietic phenotypes. *Synaptotagmin13*-MO injections produced predominant areas of blood pooling (Figure 3S). Injection of *SLC27A2-Like*-MO was even more severe as a little blood was observed. Neither observation following MO injection was attributed to defects in vascular development as the Tg (*fli-1*:*eGFP*) fluorescent pattern remained intact. In combination, the results from the vascular and hematopoietic assays of the MO screen highlight the utility of transgenic reporter strains to identify unique phenotypes that would not be detectable using other criteria such as morphology and suggest novel roles for CTT proteins in vascular and hematopoietic development.

**Figure 4:**
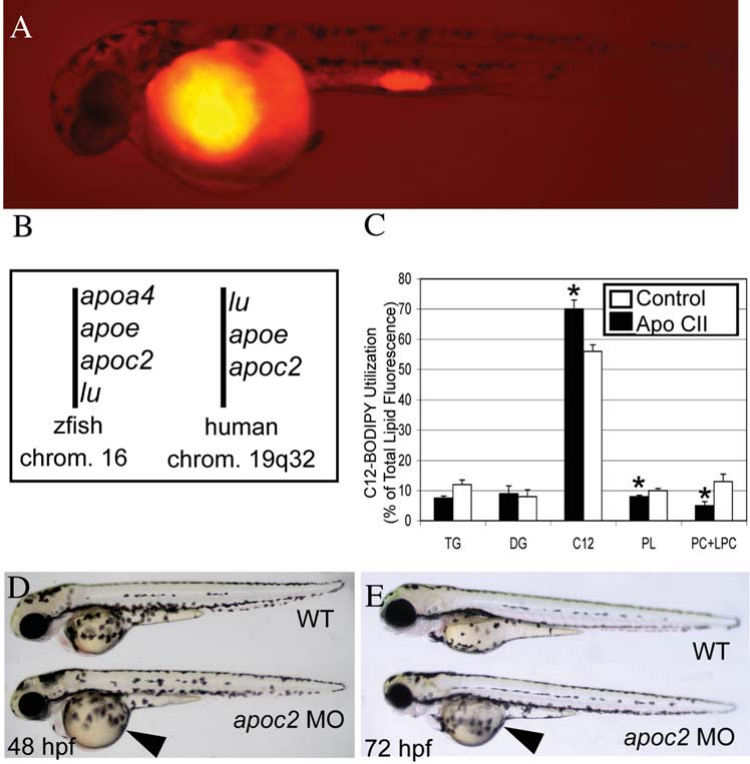
ApoC2 is required for yolk lipid procesing. Embryos at the 1–8 cell stage were initially injected with a MO of interest. At 24 hpf, a fluorescent fatty acid (BODIPY-C12) was injected into the yolk. (A) 48 hpf embryo injected with BODIPY-C12 at 24 hpf. Embryos were then kept in the dark until 72 hpf when they were scored for morphologic phenotype. Embryos (4/tube) were homogenized in 50% methanol and extracted TLC plates were then scanned to reveal triacylglycerol (TG), diacylglycerol (DG), initial substrate (C12) and phospholipids (phosphatidylcholine (PC) and lysophosphatidylcholine (LPC)). Fluorescent intensities were quantified and the total fluorescence of all lipids was determined. (B) For each MO injected, data were expressed as a percent of total lipids and compared to a phenol red control to obtain the percent of control (C) A second experiment comparing BODIPY-C12 incorporation in control and Apo2c MO injected embryos. A given experiment represents a mean of at least three individual lipid extracts with 4 embryos each. * p<0.05 (D) Syntenic analysis indicates that the zebrafish EST sequence with homology to Apoc2, is the fish ortholog of that gene. (E,F) Morphology of embryos injected with apoC2 MO. Arrowheads indicate enlarged yolk.

To screen for genes that regulate lipid processing and organ development in zebrafish, we developed an assay to study zebrafish larvae at stages before the mouth opens and swallowing begins. BODIPY-C12 (530/550) fatty acid was injected into the yolk and three day old embryos harvested for lipid extraction (Figure 4A). Although C12-BODIPY was poorly metabolized by L-cell fibroblast[7], it appears to be well incorporated into triacylglycerol, cholesterol ester, phosphatidylcholine, and phosphatidylethanolamine in zebrafish embryos. This method allowed screening of embryos with defects prior to the onset of ingestion on embryonic day 5. Morpholino injections that alter the pattern or rate of accumulation of lipid fluorescence, as well as those that produce specific alterations in larval morphology, are recorded. We found one MO that had altered BODIPY-C12 utilization. Specifically, MO-injected embryos had poorly absorbed yolk (Figure 4D & E) and lower incorporation of BODIPY-C12 into phosphatidylcholine and lysophosphatidylcholine (Figure 4C). This MO was identified as *apoC2* by syntenic analysis (Figure 4B). The poor yolk absorption was a surprising result in that the main function reported for apoC2 from studies in humans and mammalian tissue culture is the activation of lipoprotein lipase[8]. Ongoing studies are exploring if yolk absorption is dependant on lipoprotein lipase activity or whether apoC2 has other functions in the developing zebrafish embryo.

**Figure 5:**
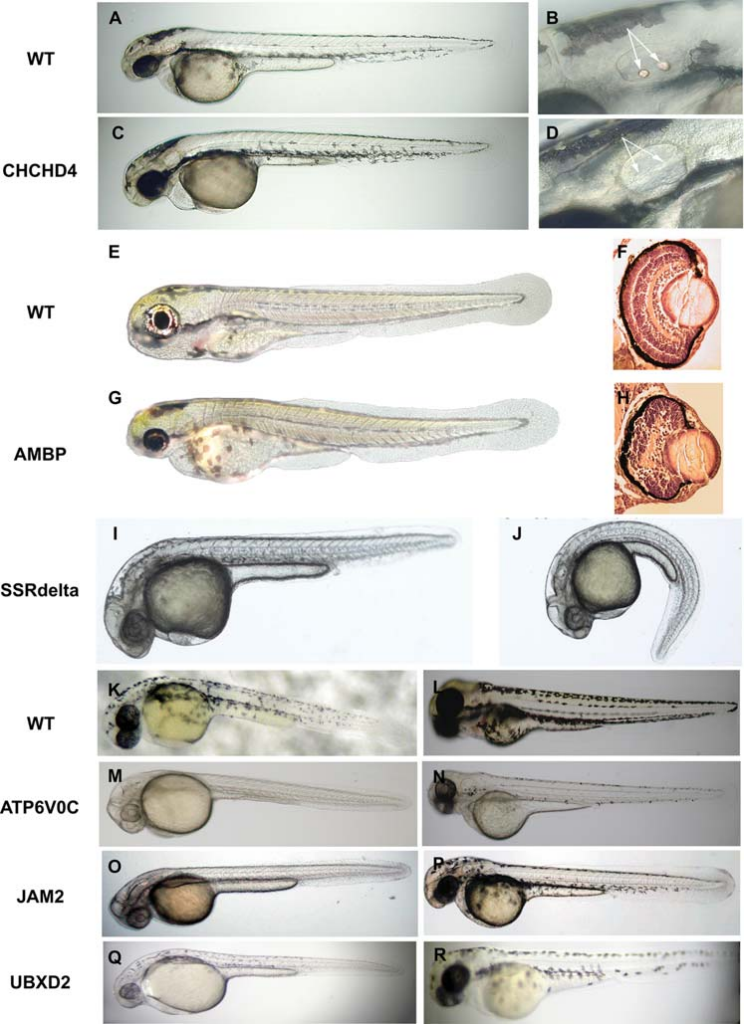
Morphological defects observed following MO inactivation of select CTT genes. (A, B) Otolith morphology observed in 2 dpf untreated embryos. (C, D) Absence of otoliths, in otherwise normal 2 dpf embryo, following injection of MO targeting CHCHD4. (B, D) Enlarged view of otic capsules; arrows denote normally formed (B) or absent (D) otoliths, respectively. (E, F) Eye morphology in 3 dpf embryos. (G, H) Abnormally small eyes observed in 3 dpf embryo following injection of MO targeting AMBP. (F, H) Enlarged view of histological sections of eye in un-affected (F) and affected (H) embryos. Note differences in both the size and tissue organization of the affected eye. (I) Wild-type morphology of 1 dpf embryo. (J) Ventral curvature phenotype observed in 1 dpf embryos injected with MO targeting SSRdelta. (K, L) Normal pigmentation observed in untreated 1 and 2 dpf embryos. Reduction in pigment observed in 1 and 2 dpf embryos, respectively, following injection of MO targeting ATP6V0C (M, N)[23], or junction adhesion molecule 2 (JAM2) (O, P), or UBX domain containing 2 (UBXD2) (Q, R)).

Morphological criteria, including standard staging and anatomical landmarks[9], were used to identify eight additional phenotypes from the MO screen. As the otic vesicle matures in 1 dpf embryos, otoliths form and are easily identifiable throughout subsequent developmental stages as shown for 2 dpf embryos in Figure 5A and 5B. Following injection of *CHCHD4*-MO, the otoliths failed to form (Figure 5C and 5D). Observations in up to 5 dpf embryos confirmed the observed defect. Development of the eye begins around the 4-somite stage with the appearance of the optic primordium and continues through development of the lens placode during the 20-somite stage providing the opportunity to assess development of the eye (Figure 5E and 5F) in the MO screen. In *AMBP*-MO injected embryos, a quantifiable decrease in the globe of the eye was observed as early as 2 dpf, and could be easily differentiated by 3 dpf (Figure 5G). Closer examination by H&E tissue staining demonstrated disrupted tissue architecture evident at 3 dpf (Figure 5H). We have also observed a phenotype involving development of the tail and trunk (Figures 5I and 5J). Following injection of *SSRdelta*-MO, a ventral curvature of the tail was characteristic of 1 dpf embryos (5J). Interestingly, this target is a protein component of the Translocon Complex, predicted to be involved in protein secretion. Consistent with the bent axis phenotype, SSRdelta is strongly expressed in the developing midline (data not shown).

The differentiation of cells in the retinal epithelium and the dorsolateral skin melanophores around the onset of the pharyngula period (24h) continue to develop into four distinct stripes on the larval trunk and tail around the late second to third day of development (Figure 5K and 5L). Changes in the development of this pigment pattern were observed in three different CTT target genes. Following injection of *ATP6V0C*-MO, very few differentiated melanophores were observed in the eye or trunk at 1 dpf (Figure 5M). Although some melanophores did appear by late 2 dpf (Figure 5N), the far fewer differentiated cells that were present appeared punctate rather than stellate in comparison with their wild-type counterparts (Figure 5L). *JAM2*-MO injection resulted in a similar lack of differentiated melanophores at 1 dpf (Figure 5O) but was characterized by morphologically normal pigmented cells at late 2 dpf that were not organized as well into the striped pattern (Figure 5P) recognizable at this time in the wild-type counterparts (Figure 5L). Injection of *UBXD2*-MO resulted in a notable delay in the onset of pigment cell differentiation at 1 dpf (Figure 5Q) compared to wild-type embryos (Figure 5K) and these embryos were distinctly abnormal at late 2 dpf (Figure 5R). Two craniofacial phenotypes, not dependent on p53-induced head cell death (unpublished observations), were also observed following injections with *HSC70*-MO and a MO targeting a novel cysteine-containing protein (Table 1). The results from the morphological screen underscore the importance of including these basic, easily observed criteria in the MO screen to identify novel regulatory functions of sensory organs and pigment cell development.

## Discussion

Although providing many mutant alleles for the study of vertebrate development, forward-genetic screens in zebrafish have drawbacks such as the large number of mutations within a gene and the considerable time required to clone and characterize these alleles. In contrast, a MO reverse-genetic approach in zebrafish embryos does not suffer from these drawbacks and has distinct advantages associated with F0 screening and *a priori* knowledge of the gene sequence. With the expanding collections of EST information and genome projects underway for multiple species, targeted discovery screening approaches using MOs as described here are now feasible. Using comparative genomic data mining strategies, we have targeted a select subset of the genome, the vertebrate secretome, by design. In addition, we focused our investigation of the role of CTT proteins in higher-order biological processes of vertebrates (such as organogenesis). Our strategy to allow partial open reading frames and improved secreted protein predictions in eukaryotic transcriptomes provides valuable tools for the analysis and annotation of eukaryotic genomes. As with many other studies, including MO screens in *X. laevis* and *C. intestinalis* [10], [11], these studies suggest TIS targeting MOs are effective inhibitors of gene expression for the study of vertebrate development provided background effects (unpublished observations) and specificity concerns[5] are addressed (such as confirming that at least two sequence independent MOs produce the same phenotype). Confirmation in at least the case of *ATP6V0C*-MO that the phenotype observed was also validated by the *hi1207*mutant of an insertional mutagenesis screen[12], further demonstrated the validity and sensitivity of the screen.

Mutagenesis work provides one estimate for the rate of visible effects due to single gene mutation in zebrafish. Saturation estimates using visible morphological phenotyping criteria suggest 2000-2400 total genes of unique function can be identified using that approach[13], [14]. Assuming the zebrafish genome includes 24-36,000 genes (numbers extrapolated from the human and fugu genome projects[15], [16], this suggests that 1 in 10 to 1 in 18 genes when mutated will yield a detectable phenotype visible during the first 5 days in development. Of those, only 30% result in developmentally ‘specific’ defects[14], suggesting that the rate at identifying biologically specific phenotypes from a random gene set is ∼2–3%.

Data from this MO screen suggests a phenotypic detection rate of ∼12% (18/150). We attribute this high discovery rate to several factors. First, some of the noted phenotypes would not have been detected using standard morphological criteria, including MOs with defects in lipid metabolism and vascular function. Second, we believe the secretome is enriched for key genes involved in regulatory and signaling function(s) and will be more likely to elicit phenotypes with regional or ‘specific’ defects. Third, translational blocking MOs are able to target both maternal and zygotic messages[1], suggesting some functions can be uncovered using MOs that would not have been detected using standard mutagenesis approaches. Finally, the ability of MOs to elicit a full range of phenotypes due to altered dosing may identify hypomorphic-like phenotypes that would be too difficult to analyze from a strong, near-null allele. We consider the current 12% detection rate to be a lower estimate of observable specific phenotypes from the screen, as additional screening will examine the morpholino collection using a variety of novel assays (such as newly generated enhancer and gene trap lines; Balciunas et al., 2004; Kawakami et al., 2004; Parinov et al., 2004) and may reveal developmental and/or functional aspects not readily visible by morphological criteria.

The 150 gene screen conducted here is too small to extrapolate to an entire genome proper, but the core observations that some phenotypes are only detectable after the use of non-visible assays suggests that current 2000 gene numbers[14] are likely underestimates. In addition, with a few notable exceptions[17]–[19], the role of the maternal genome in early development has been largely underexplored through the focus on conventional, zygotic-based genetic analyses. Developing a comprehensive dataset on the conserved vertebrate secretome, including the extant of maternal involvement in gene function, should help answer the question: How many genes are required to make a vertebrate embryo?

Assuming the vertebrate genome encodes ∼2500 conserved members in the secretome[3], this pilot study of 150 members suggests that an additional ∼250 phenotypes are yet to be uncovered after a genome-wide screen using this approach. Investigation of the current annotation associated with CTT protein phenotypes observed in this screen does not suggest any common specific functional classification associated with CTT gene. As a result, we continue to expect to see a variety of phenotypes from the ongoing screen in processes including embryonic patterning, sensory organ formation, lipid metabolism, and blood and vascular development. Evidence of the significant impact of this work is provided by our previous and continuing reports characterizing phenotypes of CTT proteins from this screen[20]–[23] and suggests a broad role of CTT proteins in developmental regulatory mechanisms. Our observations of CTT proteins in association with biological and biochemical pathways that may be uniquely vertebrate, e.g. neural crest formation and most organogenesis pathways, highlight the significant capacity of this approach to further understand clinically relevant developmental processes. In addition, molecules identified as crucial for development *in vivo* may likely serve as key substrate molecules for potential small molecule drug target intervention and for the establishment of conditions for stem cell manipulation such as in vitro organ formation. As a result there is much to be gained by the continuation of this study in understanding vertebrate development, identifying novel medical intervention targets, and ultimately improving the understanding of human genetic disease.

## Materials and Methods

### Identifying CTT proteins of the zebrafish secretome

Identification of these gene targets was expanded from a previously reported pilot analysis[2] using a defined vertebrate secretome[3]. Briefly, homology between the tentative consensus sequences (TCs) from EST information of the Institute for Genomic Research (TIGR) Zebrafish Gene Index[24] and the *H. sapiens*, *M. musculus* or *F. rubripes* protein reference secretomes (via BLASTX) was used to select a set of candidate CTT protein sequences. To enrich this set with N-terminally complete peptides, sequences were retained only if the N-termini of the homologous sequence pair aligned within a 50 amino acid threshold (homology threshold as described in Klee 2001) and/or there was a clearly predicted translation start site on the TC sequence. In cases where zebrafish EST sequences with good homology and alignment to the reference protein lack the necessary 5′UTR sequence information for MO target oligo design, we attempted to use ENSEMBL (http://www.ensembl.org, multiple build versions) zebrafish genomic data to extend the EST sequence data. The N-terminally complete peptides from selected TC sequences were then analyzed using the TargetP[25] and SignalP[26] algorithms to identify putative CTT proteins for MO design.

### MO sequence site selection and design

Utilizing the selected CTT zebrafish sequence data identified above, AMOD software[27] was used to display the identities and alignments (above) for manual confirmation and selection of the translation initiation site (TIS). Once a TIS is selected, AMOD displays all potential 25mer MO target oligos upstream of the putative start site in the zebrafish EST sequence. For each potential MO target sequence, AMOD calculates oligo-specific properties considered in design such as G, and C content. Antisense morpholino oligos are selected with 40–60% GC content, less than 37% G content, and a lack of any consecutive tri- or tetra-G nucleotide sequences. In addition, AMOD displays intra-sequence and inter-sequence homology between one or two selected oligo targets to minimize self or pair sequence homology. Acceptable oligos are selected by the user, and MO design sequence is written to an output file.

Parallel to the development of the bioinformatics pipeline to identify CTT proteins and design MOs, semi-automated filtration steps were undertaken to prioritize genes used for MO targeting. Genes lacking extensive annotation were prioritized in order to minimize overlap with work done by other zebrafish labs worldwide and genes deposited in GenBank were excluded entirely. We gave higher priority to novel genes by selecting for those sequences not possessing ENSEMBL human homologs or possessing homologs annotated as “not described” in the ENSEMBL database. Finally, we have also depreciated the value of a single protein family related to the “zebrafish egg envelope protein ZP3” that appears frequently (35 times) within the current secretome collection.

### Embryo maintenance and staging

Wild-type zebrafish were purchased from Segrest Farms (Gibsonton, FL, USA). Embryos were collected following group mating and raised at 30°C as described previously[9], [28].

### Morpholino injection

All MOs were purchased from Gene Tools, LLC (Philomath, OR, USA), prepared, and injected into 1–4 cell stage embryos as previously described[1], [28]. For sequence information see Arnold and Mabel Beckman Center for Transposon Research Morpholino Database (MODB, http://www.secretomes.umn.edu/MODB/). Initial injections of MOs were at 1.5, 3, 4.5, and 6 ng in greater than 50 wild-type, Tg (*fli-1*:*eGFP*), Tg (*gata-1*:*DsRed*), or double Tg embryos. Dosage was subsequently refined based on efficacy and toxicity profiles observed for each MO.

### Fluorescence analysis of transgenic zebrafish for vascular and hematopoietic development

GFP/DsRed expression in embryos was examined using Zeiss Axioscope 2 compound microscope (Carl Zeiss, USA) and images were captured using the Axiocam digital camera as described previously[28], [29]. MO-injected embryos were compared with uninjected controls from the same clutch at 30 and 38 hpf for visualization of DsRed blood cells and EGFP vasculature.

### BODIPY-C12 assay

Embryos are injected with a MO that targets a gene of interest as described above. At 24–30 hours post fertilization (hpf), the embryos are injected a second time with BODIPY-C12 (530/550- Invitrogen/Mol. Probes) fatty acid (approx. 0.1 ng/embryo) directly into the yolk. The embryos are stored in the dark until 72 hpf, at which time the embryos are separated into groups of four and homogenized using a bath sonicator or pestle. The lipid fraction is then subjected to TLC and analyzed with a fluorescence scanner for any perturbation of lipid processing. Immediately after phenotypic analysis, embryos (4/tube) are homogenized in 50% methanol and extracted (water∶methanol∶chloroform; 1∶1∶2; v/v). Lipid extracts are then subjected to thin layer chromatography (TLC) to determine the levels of fluorescent acyl chain incorporation. Fluorescent intensities are quantified (ImageQuant, Molecular Dynamics) and the total fluorescence of all lipids combined is determined. For each MO injected, data are expressed as a percent of total lipid fluorescence and then compared to the phenol red injected control. Prior to injection, BODIPY-C12 is purified via TLC and resuspended (10% Ethanol: 90% H_2_O) and stored for no more than 1 week. Antisense injections that alter the pattern or rate of accumulation of lipid fluorescence, as well as those that produce specific alterations in larval morphology are recorded for future analysis.

### Lipid extraction and analysis

Lipids from embryos are extracted into CHCl_3_ [30]. Neutral lipids and phospholipids are separated by TLC in heptane/isopropyl ether/acetic acid (60∶40∶4; v/v) or CHCl_3_ /methanol/40% methylamine (60∶20∶5; v/v), respectively. Triacylglycerol and phospholipids are quantified by glycerol[31] and phosphate[32] analyses, respectively. Hydrolysis products are separated by TLC in CHCl_3_/methanol/H_2_O (65∶25∶4; v/v) and the fatty acids and 1-acyl-lysophospholipid areas are scraped and extracted.

### Morphological assessment and histology

MO-injected embryos were assayed during the first three days of development using specific morphological and molecular criteria based on staging and anatomical landmarks as described previously[9]. Development of pigment was visually monitored from 24–48 hours for presence of melanophores or alterations in melanophore distribution (xanthophores and iridophores were not examined). Histological sections were fixed in 10% phosphate buffered formalin, embedded in paraffin, serial sectioned (7 micron), and stained with hemotoxylin and eosin (H&E) (Personal communication-Keith Cheng).

## References

[1] Nasevicius A, Ekker SC (2000). Effective targeted gene ‘knockdown’ in zebrafish.. Nat Genet.

[2] Klee EW, Ekker SC, Ellis LB (2001). Target selection for Danio rerio functional genomics.. Genesis.

[3] Klee EW, Carlson DF, Fahrenkrug SC, Ekker SC, Ellis LB (2004). Identifying secretomes in people, pufferfish and pigs.. Nucleic Acids Res.

[4] Summerton J (1999). Morpholino antisense oligomers: the case for an RNase H-independent structural type.. Biochim Biophys Acta.

[5] Ekker SC (2004). Nonconventional antisense in zebrafish for functional genomics applications.. Methods Cell Biol.

[6] Eckfeldt CE, Mendenhall EM, Flynn CM, Wang TF, Pickart MA (2005). Functional analysis of human hematopoietic stem cell gene expression using zebrafish.. PLoS Biol.

[7] Huang H, Starodub O, McIntosh A, Kier AB, Schroeder F (2002). Liver fatty acid-binding protein targets fatty acids to the nucleus. Real time confocal and multiphoton fluorescence imaging in living cells.. J Biol Chem.

[8] Shen Y, Lookene A, Nilsson S, Olivecrona G (2002). Functional analyses of human apolipoprotein CII by site-directed mutagenesis: identification of residues important for activation of lipoprotein lipase.. J Biol Chem.

[9] Kimmel CB, Ballard WW, Kimmel SR, Ullmann B, Schilling TF (1995). Stages of embryonic development of the zebrafish.. Dev Dyn.

[10] Kenwrick S, Amaya E, Papalopulu N (2004). Pilot morpholino screen in Xenopus tropicalis identifies a novel gene involved in head development.. Dev Dyn.

[11] Yamada L, Shoguchi E, Wada S, Kobayashi K, Mochizuki Y (2003). Morpholino-based gene knockdown screen of novel genes with developmental function in Ciona intestinalis.. Development.

[12] Golling G, Amsterdam A, Sun Z, Antonelli M, Maldonado E (2002). Insertional mutagenesis in zebrafish rapidly identifies genes essential for early vertebrate development.. Nat Genet.

[13] Driever W, Solnica-Krezel L, Schier AF, Neuhauss SC, Malicki J (1996). A genetic screen for mutations affecting embryogenesis in zebrafish.. Development.

[14] Amsterdam A, Hopkins N (2006). Mutagenesis strategies in zebrafish for identifying genes involved in development and disease.. Trends Genet.

[15] Aparicio S, Chapman J, Stupka E, Putnam N, Chia JM (2002). Whole-genome shotgun assembly and analysis of the genome of Fugu rubripes.. Science.

[16] Venter JC, Adams MD, Myers EW, Li PW, Mural RJ (2001). The sequence of the human genome.. Science.

[17] Dosch R, Wagner DS, Mintzer KA, Runke G, Wiemelt AP (2004). Maternal control of vertebrate development before the midblastula transition: mutants from the zebrafish I.. Dev Cell.

[18] Pelegri F, Schulte-Merker S (1999). A gynogenesis-based screen for maternal-effect genes in the zebrafish, Danio rerio.. Methods Cell Biol.

[19] Wagner DS, Dosch R, Mintzer KA, Wiemelt AP, Mullins MC (2004). Maternal control of development at the midblastula transition and beyond: mutants from the zebrafish II.. Dev Cell.

[20] Chen E, Hermanson S, Ekker SC (2004). Syndecan-2 is essential for angiogenic sprouting during zebrafish development.. Blood.

[21] Chen E, Stringer SE, Rusch MA, Selleck SB, Ekker SC (2005). A unique role for 6-O sulfation modification in zebrafish vascular development.. Dev Biol.

[22] Chen E, Larson JD, Ekker SC (2006). Functional analysis of zebrafish microfibril-associated glycoprotein-1 (Magp1) in vivo reveals roles for microfibrils in vascular development and function.. Blood.

[23] Pickart MA, Sivasubbu S, Nielsen AL, Shriram S, King RA (2004). Functional genomics tools for the analysis of zebrafish pigment.. Pigment Cell Res.

[24] Liang F, Holt I, Pertea G, Karamycheva S, Salzberg SL (2000). An optimized protocol for analysis of EST sequences.. Nucleic Acids Res.

[25] Emanuelsson O, Nielsen H, Brunak S, von Heijne G (2000). Predicting subcellular localization of proteins based on their N-terminal amino acid sequence.. J Mol Biol.

[26] Bendtsen JD, Nielsen H, von Heijne G, Brunak S (2004). Improved prediction of signal peptides: SignalP 3.0.. J Mol Biol.

[27] Klee EW, Shim KJ, Pickart MA, Ekker SC, Ellis LB (2005). AMOD: a morpholino oligonucleotide selection tool.. Nucleic Acids Res.

[28] Hermanson S, Davidson AE, Sivasubbu S, Balciunas D, Ekker SC (2004). Sleeping Beauty transposon for efficient gene delivery.. Methods Cell Biol.

[29] Davidson AE, Balciunas D, Mohn D, Shaffer J, Hermanson S (2003). Efficient gene delivery and gene expression in zebrafish using the Sleeping Beauty transposon.. Dev Biol.

[30] Bligh EG, Dyer WJ (1959). A rapid method of total lipid extraction and purification.. Can J Biochem Physiol.

[31] Fletcher MJ (1968). A colorimetric method for estimating serum triglycerides.. Clin Chim Acta.

[32] Chen PSJ, Toribara TY, Warner H (1956). Microdetermination of phosphorus.. Anal Chem.

